# Epitome of the Region—Regional Nostalgia Design Based on Digital Twins

**DOI:** 10.3390/bs15010012

**Published:** 2024-12-27

**Authors:** Liling Chen, Yicong Song, Xiaojing Niu, Xin Luan, Liu Yang, Shengfeng Qin

**Affiliations:** 1 School of Mechanical Engineering, Northwestern Polytechnical University, Xi’an 710072, China; 2 Smart Design Lab, School of Design, Northumbria University, Newcastle upon Tyne NE1 8ST, UK

**Keywords:** nostalgia design, digital twin, regional public memories, scene model, design for emotional wellbeing

## Abstract

Nostalgic scenes can trigger nostalgia to a considerable extent and can be effectively used as a nostalgic trigger that contributes to the psychological comfort of the elderly and immigrant populations, but a design system has not been adequately studied. Therefore, the design principles and digital twin (DT) design system of nostalgic scenes is proposed in this study. It focuses on the construction of a nostalgic scene DT model based on the system of system (SoS) theory. Nostalgic scenes related to farm work are selected and photos of this DT model from a particular perspective are generated for presentation. Co-occurrence analysis is used to verify the correlation between elements within the scene. We invited two groups of residents in Xi’an, the regional group and the non-regional group, a total of 68 people, as participants to rate three photos with different degrees of design on the Likert scale. The results of data analysis show that systematic and well-composed nostalgic scene images, which incorporate relevant elements, are more likely to evoke participants’ nostalgic emotions than ones without those elements mentioned above. Likewise, a series of nostalgic scene images spanning various periods can stimulate participants’ nostalgic emotions more effectively than a single image. Furthermore, region-specific nostalgic scene images that resonate with participants sharing similar lifestyles can trigger their nostalgic feelings more effectively. The digital twin model of the nostalgia scene contains multi-source data, which can be dynamically visualised to represent regional nostalgic experiences. The design system can be used to design nostalgic scenes to improve emotional health, social bonding, tourism, and sustainable urban and rural development.

## 1. Introduction

Rapid digitisation and urbanisation in our society have contributed to technology phobia, unfamiliarity, and loss of memories in parts of the population (Gonzaga, 2019). Social and family obligations and work pressures cause young people to consider a carefree childhood as an optimal time. The elderly also often missed having contact with society when they were young. Based on these two phenomena, nostalgia helps vulnerable population groups establish a connection between the past and present, which strengthens self-confidence and life satisfaction (Raymond, 2013).

Nostalgia is a universal psychological phenomenon that occurs regardless of gender, age, status, or ethnic background and is a self-relevant emotion enabling the free fictionalisation of imaginary worlds (Sedikides et al., 2004). As more of our lives are replaced by mass production, mass media, and mass consumption, the past also becomes a mass-produced and consumed commodity (Wagner, 2018). Due to the regionalism and abstractness of nostalgic emotion (Kim et al., 2019b), the cost of nostalgia design is too high for the minority, and nostalgia design is too monotonous for the public. For this reason, the study of the systematic theory for the existing design of nostalgic scenes is insufficient. Simple static sensory stimulation such as old photos or old items as carriers is adopted directly in existing research on nostalgia to trigger nostalgia for the objects of study. However, in addition to simple old items, music, or food, narrative scenes, such as a life period in the distant past that can no longer be experienced, show stronger effects on triggering nostalgia. 

The research objective is to develop a comprehensive design method capable of eliciting nostalgic emotions among subjects more effectively than mere object carriers and to validate the efficacy of this approach. To trigger nostalgia of the vulnerable groups in the city and to promote their mental health, (1) this study summarises the design principles of nostalgic scenes (regional, full life-cycle, and systematic), and (2) this study proposes the construction approach of a digital twin (DT) system framework for nostalgic scenes, dividing nostalgic scenes based on the system of system (SoS) theory and providing the main perspective of the DT model in the form of photos. (3) This study tests the validity of the nostalgic scene DT model as a nostalgic trigger through questionnaire analysis and comparison. The proposed DT system consists of three parts: the physical, the DT model, and the application. The DT model, serving as the primary record of nostalgic life, contains three layers: nostalgic life, nostalgic scenes, and nostalgic FEATUREs. Among these three aspects, nostalgic scenes reflect part of the nostalgic life from different perspectives and nostalgic FEATUREs refer to events and concrete objects in the scenes that participants recall. These FEATUREs, which are combined to form the nostalgic scenes, can indicate changes in the scenes. The established DT model of nostalgic scenes can reflect historical changes and aid in the prediction of evolutionary trends. 

The key contributions of this study are as follows:

(1) Proposing a DT system framework for nostalgia design based on regional nostalgic scenes and describing the construction of the nostalgic scene DT model based on SoS theory, where photos of nostalgic scenes are generated to express the main perspective of their DT model.
(2) Demonstrating the expected nostalgia design results of a regional, full-life-cycle and systematic nostalgic scene DT model, with an example from rural northern China, and obtaining nostalgic FEATUREs through questionnaire interviews and conducting co-occurrence analysis in the classic text records to validate the coherence of the selected nostalgic FEATUREs. Employing images as carriers of nostalgic scenes, the subjects were invited to participate in experiments and provide evaluations.

This research is organised as follows. Section 2 presents related work on nostalgia emotions, nostalgia design practices, and influences. Section 3 introduces the theoretical foundations of nostalgia design and presents the design framework and modelling steps for the DT. Section 4 applies the framework to rural areas in developing countries, using two communities in Shaanxi, China, associated with rural life as examples. The researchers invited residents to participate in design experiments. They created photos from the perspective of a nostalgic scene DT model (hereinafter referred to as the photo) to reflect their digital twin model and compared five sets of questionnaires to prove the feasibility of the system and the validity of the design principles. Section 5 discusses the social implications of the DT system and design principles for nostalgia design. At the end, conclusions and suggestions for future research are shown in Section 6.

## 2. Related Work

### 2.1. Nostalgia as a Positive Emotion

The word ‘nostalgia’ combines two Greek words: ‘nostos’ (return) and ‘algia’ (agony), connoting longing for home, family, and friends (Alasmari, 2023). The Oxford Dictionary defines nostalgia as “a feeling of longing for the past, usually a pleasant personal experience of a period or place” (Wagner, 2018). According to Davis (1979), nostalgia arises mainly from people’s contempt for and dissatisfaction with the present. Additionally, people tend to have a distorted view of the past, describing it as better than the present (Fox, 1981).

Nostalgia is a mental phenomenon valued and explored across various fields of social science, including history (Moniz, 2021), psychology (Leunissen, 2023), sociology (Hepper et al., 2021), the humanities (Shi et al., 2021), and environmental psychology (Kim et al., 2019b). Nostalgia touches on people’s experiences and culture, stimulating wellbeing among those who recall autobiographical memories (Hepper et al., 2021). Nostalgia can be spontaneously evoked to buffer the psychological threat of limited time horizons and facilitate the maintenance of psychological wellbeing across the adult life span (Hepper et al., 2021). Nostalgia also enhances positive psychological states such as self-continuity, self-esteem, optimism, inspiration, and social connectedness (Wildschut & Sedikides, 2023). Studies showed that the higher the intensity of the experience felt, the longer the memory will last and the stronger the emotion of nostalgia (Liu et al., 2010). People who are sensitive to dispositional nostalgia derive more psychological benefits from nostalgic experience (Layous et al., 2021).

Nostalgia is a highly social emotion that also enhances group identity and cohesion and provides a sense of connectedness with others (Juhl & Biskas, 2023). Nostalgia can be divided into individual nostalgia and collective nostalgia, with individual nostalgia associated with individual experiences and collective nostalgia associated with shared experiences and cultural memories. Nostalgia is more common among people who have experienced hardship together. When individuals consider themselves part of a group, the events and objects associated with that group acquire an emotional significance for the individual over time (Mackie & Smith, 1998).

### 2.2. Nostalgic Trigger

Nostalgic triggers can be divided into external and internal (Godbole et al., 2006), with external triggers including sensory stimulation, environment, storytelling, and experience and internal triggers including recall and self-imagination. Nostalgic triggers are achieved by presenting objects, events, and scenes, among others, to participants. Researchers utilised different nostalgic triggers to quantify the participants’ nostalgic attitudes through questionnaires and physiological data, such as EEGs (Hungenberg et al., 2020).

Sensory stimulation such as sight, hearing, smell, and taste can trigger nostalgia. Researchers utilised carriers such as nostalgic photos (Shi et al., 2021), logos (Nam et al., 2016), archaeological productions (Knight, 2023), old artifacts (Christou et al., 2018), popular music (Barrett et al., 2010), different scented perfumes (Reid et al., 2015), and foods (H. Song et al., 2021) to stimulate the senses and evoke nostalgia. The authors (Wagner, 2018) narrate the story of a British potato chip company using nostalgic old black-and-white photographs as packaging, evoking general consumer nostalgia for small-scale, craft-based food production. Studies have demonstrated that nostalgia associated with different sensory stimulation has similar effects on psychological function (Green et al., 2023). Nostalgia memories triggered by smell are more emotional and include more relevant details of events (Dimitriadou et al., 2019), whereas nostalgia memories triggered by taste and smell are particularly self-relevant, evocative, and familiar (Green et al., 2023).

Environment, storytelling, and experience are multiple sensory stimulations, and experiences that incorporate multiple sensory factors may help individuals more fully immerse in nostalgic reverie. A pavilion at the Greek Archives in South Africa, as described in (Moniz, 2021), reconstructs memories and re-creates archival material through digitisation, sound, texture, projection, and colour. The environmental stimulation of sociability is a prominent trigger of nostalgia, and specific decorations, layouts, and materials in an environment can also trigger nostalgia. For example, visiting historical sites (Kim et al., 2019a), museums (Anderson et al., 2021), and theme parks (Oh & Kim, 2020) can evoke memories in visitors. Guan et al. (Guan et al., 2019) researched Yuanjiacun in Shaanxi, China. In a successful commercial snack street, snack bars sell traditional food as a carrier for nostalgia; the attire of shopkeepers and the decoration of stores are well suited to the historical countryside, satisfying the imagination and nostalgia of urban tourists. 

People often make emotional connections between the self and experiences. For example, classic baseball games (Hungenberg et al., 2020), nostalgic movies (Kim et al., 2019b), nostalgic animations (Lin et al., 2021), and classic movie characters (Hosany et al., 2020) can all bring back memories of previous experiences. Virtual display technology and digitisation technology can complement immersive experiences, effectively attracting visitors and promoting activities (Oh & Kong, 2021).

The internal triggers are recall and interview tasks, which ask participants to recall or reflect on past experiences to evoke nostalgia. Reid et al. asked participants to visualise eating a nostalgic food and consumed 12 flavour samples to compare two that triggered nostalgia (Reid et al., 2022). Moreover, not only in research, scholars across culture and history fields from numerous countries have depicted uplifting and heart-warming past scenes for the public, including agricultural landscapes in Japan’s Showa Era (Davidson, 2022), religious celebrations in Guadalupe (Ortiz, 2022), the Chinese literati’s root search due to urban–rural dichotomies (Han, 2021), and British countryside manors during epidemics (Kay & Wood, 2022).

### 2.3. Application of Nostalgia

Quantitative research shows the relationship between nostalgia and emotion, sociology, advertising, and so on, reporting the multidimensional characteristics of nostalgia and its application value in different fields such as marketing (Weingarten & Wei, 2023), tourism, and safety.

Nostalgia can establish a bond with consumers and retain customer loyalty, but only among consumers with past exposure or connection with the brand (Weingarten & Wei, 2023). Nostalgic advertising and marketing strategies can also be effective in attracting consumers and enhancing brand value. Consumers with high nostalgia proneness are more likely to buy nostalgic products and brands and recommend them to others (Nam et al., 2016). It was found that a nostalgic Starbucks ad (e.g., 1971 Starbucks logo), relative to a non-nostalgic Starbucks ad (e.g., current Starbucks logo), was more likely to induce purchase intentions (Nam et al., 2016). Stakeholders can emphasise the characteristics of consistency and continuity of goods to foster authenticity perception, which can attract consumers with active experience (Cho et al., 2021). In Wallner’s research, refurbished products that apply the design characteristics from the past are considered more durable and aesthetically appealing because they evoke associations with the good quality of the past (Wallner et al., 2020).

Nostalgic memory has a strong established relationship with regions and scenes, nostalgia-induced place attachment, which has a positive effect on tourists’ revisit intention. Place attachment may be induced by a classic film (Kim et al., 2019b), baseball game (Cho, 2021), study abroad experience (Cho et al., 2021), and so on, driving travelers to revisit those destinations to have nostalgic positive memories and even stimulate the willingness to buy souvenirs. Ewing (2024) found that within London’s diaspora communities, vendors sell Nollywood DVDs as physical commodities from their homeland, triggering sensations of nostalgia, and preserving Black African cultural identity abroad.

Nostalgic experiences are dynamic and subject to temporal variations; correspondingly, the expressions of nostalgic triggers should ideally be coherent and dynamically evolving. Song et al.’s research (S. Song et al., 2024) shows that end temporal landmarks trigger feelings of nostalgia, which leads to nostalgic consumption through the need to belong. Marketers may use the signs of end temporal landmarks to increase consumers’ nostalgia, which, in turn, will enhance consumers’ need to belong and thus lead to the purchasing and consumption of nostalgic products.

Scholars use rich elements, complete storytelling, realistic scene rendering, and multi-dimensional sensory experience to enhance the user’s nostalgic experience, which can be summarized as systematic design. Wang et al. designed two nostalgia devices: a game machine simulating the 1900s planting and harvesting process and a game turntable playing nostalgic music with an old-fashioned appearance (Wang & Huang, 2023; Wang et al., 2020). This aimed to encourage the elderly to exercise, engage with technology, and increase interest in sport. Taking traditional-village digital museum (TVDM) tourism as the research object, Sang (2024) strengthened the tourists’ sense of nostalgia and stimulated their ritualized nostalgic behavior through embodied interaction with virtual reality. Huang et al. (2024) found that compared with the text-reading VR experience, nostalgic storytelling VR resulted in significantly higher hedonic and greater state nostalgia.

Nostalgia can be used as good social welfare and positive induction, caring for vulnerable groups. The nostalgic reminiscence approach has been experimented with and reported as an alternative treatment that can activate the minds of elderly and dementia patients (Tsao et al., 2019). This approach may be perfect for providing comfort in the face of death and loneliness threats, as it will temporarily wrap people in the joy of a lighter past (Christou et al., 2018). Cho et al. (2023) collected 302 responses from participants who had engaged in sport activities before the coronavirus disease (COVID-19) lockdown period. The findings showed that nostalgia positively affected the self-regulation of sport and subjective well-being. Simpson et al. (2024) discovered a significant correlation between food-evoked nostalgia and mood, indicating that nostalgic foods may mitigate negative mood states, leading to enhanced positive mood states and an improved quality of life for individuals with low mood states. Cho et al. (2024) found that nostalgia plays a key role in motivating Singaporean adults to overcome barriers to participation in sports, reconnecting them with positive past experiences and fostering a desire to participate in physical activity.

### 2.4. Design Principles for Nostalgic Scenes

Scenes are a significant factor in raising nostalgia. Experimental studies often use single, simple, existing objects as carriers to explore the effects of nostalgia and the differences in individuals of different ages and cultural backgrounds. However, in order to serve the mental health of the elderly and strangers, the optimal choice of nostalgic triggers is rich environments or scenes, as they can provide multi-sensory stimulation. A nostalgic scene can reproduce a nostalgic life that has happened in the past and is difficult to recur due to time or space. 

Multiple resources are integrated and organised in the design of nostalgic scenes. The design of nostalgic scenes can be realised by referring to historical buildings or it can be recreated by simulating the site of the past. For example, Tsao et al. integrated AR/VR three-dimensional models and video/audio interaction along with other technologies to decorate ancient building models with nostalgic images, but the images were incongruent with the appearance of the ancient buildings. With that being said, there are no adequate studies on the design practice of nostalgic scenes. Hence, this study summarises and proposes three design principles of the nostalgic scenes: regional, full life-cycle, and systematic.

#### 2.4.1. Regional

Each group, be it a community, a village, or a family, forms an emotional space containing public memory based on shared territory (Bartlett, 1932). For example, Hong Kong films have influenced the youth memories of most residents in Hong Kong and Taipei (Kim et al., 2019b). Syrian refugees residing in Saudi Arabia are generally less optimistic about their memories of the past (Hepper et al., 2021). When a generation dies out, relevant parts of their memories can be preserved as history, making them publicly accessible.

**Figure 1:**
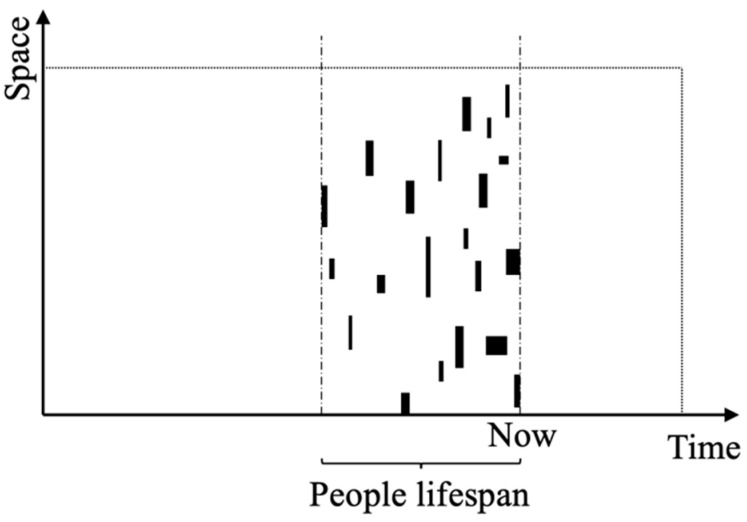
Distribution of public memory in time and space. Source: self-elaboration.

Figure 1 further explains public memory. Every public memory worldwide can be represented abstractly as a square, with its length and width representing the spatial spread and the temporal duration, respectively. Public memory, which pertains to specific events from the past and certain regions, exists within the temporal and spatial boundaries of the lifespans of the individuals currently living in society. For instance, people in one place may be keen on watching Hong Kong movies, which becomes a public memory. In another place, people may have a particular sacrificial activity that is unique to their village. These situations, which may vary in their temporal duration and spatial extent, impact different population groups born in various periods. However, the recreation of public memory through nostalgic scenes can evoke emotions associated with personal experiences in a specific region. Past experiences can cause them to develop a place attachment to the restored scene, which triggers their nostalgia. A public memory with a larger “space” affects the nostalgia of residents in a larger area, and a public memory with a longer “time” has a more significant and profound effect on residents’ nostalgia. Therefore, the design of nostalgic scenes is suggested to be regional, and consideration should be given to the question of the public memory of which group is reflected by nostalgic scenes.

#### 2.4.2. Full and Moving Life Cycle

The modern concept of time is characterised by irreversibility (Simon, 2005). This definition shows that the future cannot provide what the past has given us, but the past can be the raw material underpinning the imagination of the future. Nostalgia is thus not only retrospective, in that it temporarily escapes reality or presents the past in a fixed form, but also future-oriented, because it inspires us to remember the past and recover history in a certain way. Shi et al. found that people were able to perceive stronger and more complex emotions in older nostalgic photos (Shi et al., 2021). Therefore, the design of nostalgic scenes is suggested to be full life-cycle.

#### 2.4.3. Systemic

The more realistic, dynamic, multi-sensory, and multi-modal the scenes are, the more easily nostalgia is triggered in users (Kelley et al., 2022). A nostalgic scene is systematic; it is not only a combination of objects and events, but each scene also has a systematic correlation.

Elements in the nostalgic scenes are correlated. Nostalgic scenes contain objects and events, such as tangible heritage, old items, or intangible customs and legends, which can be visually represented. The systematicness of a nostalgic scene is reflected in the close relation between objects and events which conform to the same context and background. The more systematic, realistic, and multisensory the nostalgic scenes are, the more these factors can evoke the user’s nostalgia. For example, the elements that viewers focus on during baseball games are players, teams, music, stadiums, norms, and fans, and their interactions trigger viewers’ nostalgia for baseball games (Hungenberg et al., 2020). By the same token, the special effects, movie stars, and fighting in Hong Kong films are core elements and can arouse recollection of nostalgic scenes (Kim et al., 2019a).

**Figure 2:**
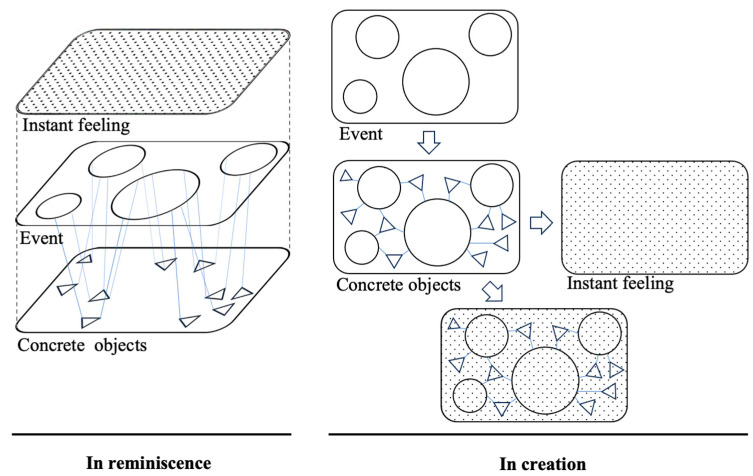
Three levels of spatial elements in nostalgic scenes. Source: self-elaboration.

The artistic presentation form of nostalgic scenes requires a mastery of common spiritual needs at the basic level. The combination and composition of spatial elements are realised at three levels. The relationship between the three levels is summarised abstractly in Figure 2.

The first level is instant feelings. It is the evaluation that immediately comes to mind when one recalls a particular scene—the socio-biographical memory of poverty or leisure, shown as dots in Figure 2. The second level is the event. It is the real experience, which is represented by circles in Figure 2. The emotional perception precisely arises from the event, and the atmosphere is created by the construction of the environment, which relies on physical representation (Hirst et al., 2018). The third level is the concrete objects in the scene. They are related to primary events and represented as triangles in Figure 2. The concrete objects can give participants clues about the settings and atmosphere, which can influence their emotions.

As shown in Figure 2, in reminiscence, the acquisition of instant feelings is prioritised and the most apparent, followed by recognising and noticing the main events in the scene, and then noticing concrete objects. The scene is akin to a story containing characters, objects, the environment, and events. In the design and creation of the scene, emphasis on the event should be prioritised, followed by the concrete objects matching the event. As mentioned above, instant feelings refer to the atmosphere created by major events and concrete objects, with each scene primarily conveying one type of instant feeling.

## 3. Materials and Methods

**Figure 3:**
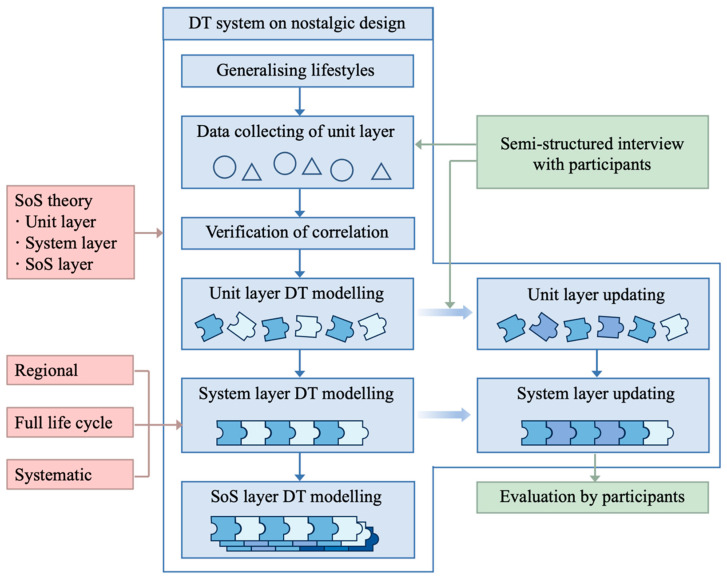
Proposed research framework.

In conformity with design principles, this paper proposes a design system of regional nostalgic scenes based on digital twins (DTs) and discusses its implementation steps. This paper focuses on constructing a DT model, with photos of nostalgic scenes serving as the representational format of the model, suggesting that photos are one perspective to view DT model expression. As shown in Figure 3, this study divides the nostalgic scenes based on SoS theory and constructs the DT model of the nostalgic scenes, which consist of units referencing Figure 2. The units can be events and objects, which are defined as FEATUREs in this study. The objects and events in the nostalgic scenes can be identified through semi-structured interviews, and the systematicness of the nostalgic scenes can be verified through co-occurrence analysis. When the DT model of nostalgic scenes is used as psychotherapy, it is mainly represented visually. Therefore, generative AI is used as a design tool to visualise the main perspective of nostalgic scenes. Finally, a case study using the questionnaire survey method is conducted to validate the proposed design system of nostalgic scenes.

### 3.1. DT System Framework for the Regional Nostalgic Scene Design

The DT concept, launched by Michael Grieves in 2003, was initially used in the military and aerospace but has since expanded. DTs featuring traceability integrate various technologies (Grieves, 2022), which enables real mapping of DTs to their physical entities and reflects real-time physical states. DTs can manage, plan, monitor, and predict changes through applications. Three-dimensional models of concrete objects in virtual scenes and recordings of event activity, among others, provide data for DT research, enabling systematic, full-life-cycle nostalgia design.

**Figure 4:**
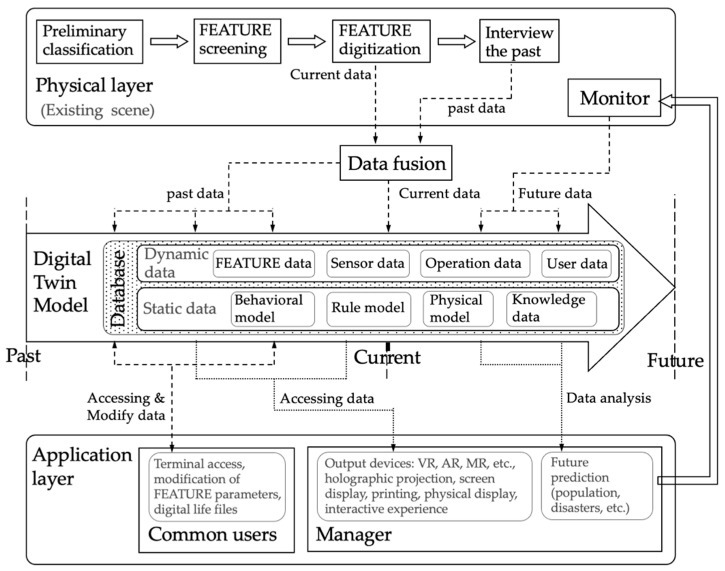
DT system for regional nostalgia design. Source: self-elaboration.

Figure 4 explains the DT system framework for regional nostalgic scenes proposed in this study. It encompasses the full life-cycle of a nostalgic scene and consists of three parts: the physical layer, the DT model, and the application layer.

At the physical layer, physical space as a typical historical site evokes nostalgia in the public and exhibits several phases of time-varying physical forms. Decades of years have witnessed the evolution, and even possible destruction and disappearance, of these objects that yet fill the memory gaps of a local population. Nostalgic FEATURE information and life-cycle changes (past and future) stem from the physical layer. FEATUREs serve as units to construct nostalgic scenes reductively and provide a complete description that reflects a perspective on nostalgic life.

In the DT model, a virtual scene model serves as a carrier, whereby 3D models of updatable FEATUREs are meaningfully placed in the scene model to reproduce a physical space. The information displayed at any point in time consists of the static data of FEATUREs and the dynamic data at that time, presented in the form of a geometric model that reflects attributes such as shape, size, and even material. Static data includes behavioural models, rule models, physical models, and knowledge data. The physical and behavioural models reflect the FEATURE’s internal properties and mechanisms (principles), and they are mainly used for back-end simulations and calculations. The rule model, which refers to constraints or laws within these three models, restricts the geometric, physical, and behavioural models. Knowledge data are collected from interviews with residents and reflect geographical preferences, taboos, and other non-uniform knowledge (e.g., two FEATUREs cannot coexist). Dynamic data contain FEATUREs, sensors, operations, and user data of the virtual scene. FEATURE data record changes in physical and geometric models, saving different physical states of FEATUREs at different timestamps. Additionally, the sensor data reflect FEATURE changes from long-term monitoring and operation and user data reflect the operational history of this DT system in the application. This study focuses on creating DT models for nostalgic scenes. However, the collection of sensors, operations, and user data is beyond its scope.

The application layer aims to provide application services for managers and ordinary users. The nostalgic scene DT model can be visualised to show the physical space at different times. The DT system mainly serves as a regional public memory in museums and other cultural institutions. Managers of museums use output devices to provide visitors with an experience and remind them of the emotional space they are missing.

### 3.2. SoS Theory

**Figure 5:**
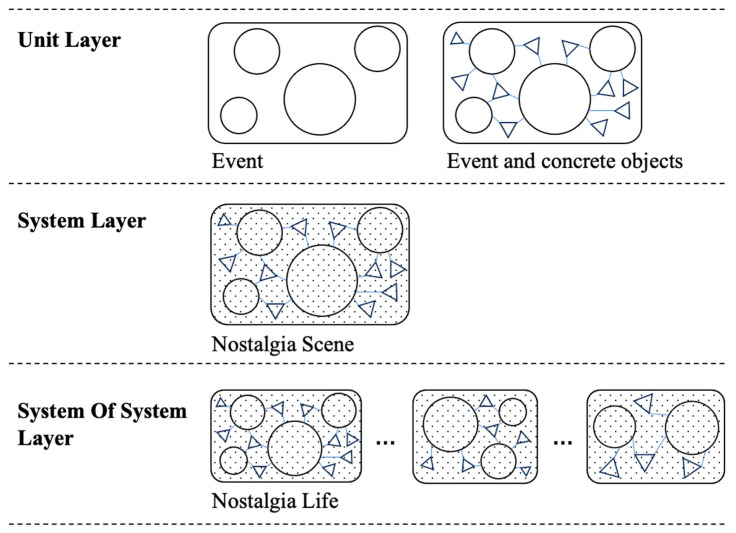
Three layers of the DT model. Source: self-elaboration.

As shown in Figure 5, the DT model of nostalgic scenes can be divided into three layers: unit, system, and SoS (Zhang et al., 2022), which correspond to the three levels of spatial elements in nostalgic life. The unit layer is characterised by indivisibility, which refers to all the FEATUREs in nostalgic scenes, including events and concrete objects. The system layer is the nostalgic scenes generated by the combination of FEATUREs, encompassing the entire historical cycle. Various nostalgic scenes converge to reflect people’s nostalgic lives from multiple perspectives, which are concluded in the SoS layer. The DT model of each scene represents a separate system, while the whole nostalgic life forms the SoS with strict hierarchical relationships. Further, the DT model of nostalgic life represents the digitised record of an entire lifespan.

The modelling process for SoS-layer DTs can follow the sequence of complexity from the simplest to the most complex by using a modular approach based on causality. In other words, the DT model of the unit layer should be prioritised, and then the DT model of the system layer should be built sequentially. Finally, the SoS DT model can be established by connecting the DT model of the system layer and integrating the key FEATUREs into an SoS (Bickford et al., 2020).

### 3.3. Construction of DT Model

The initial construction of the DT model of nostalgic scenes needs to be combined with the models of the unit layer, and its systematicness needs to be verified. With the change in a nostalgic scene over time within one life cycle, the update of the nostalgic scene model can be observed in the FEATURE model update.

Before digitisation, regional nostalgic scenes require the researcher to generalise lifestyles and summarise universal FEATUREs that are related to each group. Various parameters, such as economic development, ecological environment, and fire performance, to name a few, are proposed by scholars to establish multi-dimensional evaluation systems. It is an effective way to generalise lifestyles by referring to the historical, geological, and other characteristics of the region.

#### 3.3.1. Selecting FEATUREs and Unit Layer Modelling

Combining FEATUREs to compose a nostalgic scene corresponds to the selection and digitisation of three levels of spatial elements. The first consideration is the events and then the concrete objects that match those events. Using stakeholder insights to filter the appropriate FEATUREs (events and concrete objects) can reduce the overall cost of generating nostalgic scenes. Additionally, FEATUREs can be extracted from ancient websites, digital archives, and observational records (Krol & Zdonek, 2022). The selection of FEATUREs should follow the principle of being diversified, multi-sensory, and capable of better restoring the real past since a single FEATURE is insufficient to inspire nostalgia in residents (Spence, 2020).

Most of the FEATUREs are landscapes, architecture, and old items, mainly restored via digital 3D modelling, which enables them to reflect their structures, materials, or layouts. Character modelling focuses on particular movements, clothing, and speech habits, which are more practical for an elderly person or a child than a middle-aged person (Toppins, 2022). The digital reproduction of intangible FEATUREs, such as traditional ritual performances and experiential activities, relies on characters’ movement sequences and interactions with physical props, stages, and venues. Motion capture technology can form point cloud models of the character’s action sequences and automatically model them into 3D animations (Zabulis et al., 2020). Circular demonstration of multiple action sequences is the dynamic unit layer model of events.

#### 3.3.2. Verification of Correlation Between FEATUREs

Co-occurrence analysis is a common method of text mining, which reveals the correlation between words by studying the frequency of co-occurrence of words or keywords in a text. FEATUREs are collected through interviews with stakeholders, and co-occurrence analysis of FEATUREs extracted from authoritative historical records can verify whether the correlation between the collected FEATUREs is sufficient. Such efforts can help prove the systematicness of the scenes that contain FEATUREs (Dong & Li, 2022). FEATUREs with high co-occurrence frequency are considered to be in the same context due to semantic relevance or functional association, and FEATUREs with high frequency are considered to be more important (Jungblut & Zakarevičiūtė, 2019).

#### 3.3.3. System Layer Modelling

Nostalgic scenes are ideally designed to enhance the impact of nostalgia by collecting multiple nostalgic FEATUREs. The 3D DT models of multiple FEATUREs are combined and arranged within the digital space, following the rule model and knowledge data, to form a structured 3D model of the scene, referred to as the DT model of nostalgia scenes. Many events or activities are based on specific scenes and involving semantic scene knowledge (Zhou & Liu, 2014). For example, the distance and direction relationship between objects is localised according to the rule model and knowledge data. In addition, specific colours and materials give a semantic tendency to nostalgia.

#### 3.3.4. Data Updating and Full Life-Cycle

The DT model of a nostalgic scene is not only an instantaneous geometric model but also one that encompasses the full life-cycle of the scene in the time dimension. For example, a FEATURE was replaced or its size changed due to logical relationship updates, which should be recorded. And these changes can be reflected in the update of the scene without the effort to redesign the scene. Changes in FEATUREs can be gained from the memories of individuals who used to experience them or from historical records. In addition to manual updates, AI technology can also assist to automate updating the scene. The automatic update and reflecting of FEATUREs can prevent the nostalgic scenes from being redesigned.

Model updating provides a chance to achieve realism, dynamism, and historical changes in the scene model. Additionally, the appearance of the existing nostalgic scenes is based on past data, and future evolution could be updated in real time from current state data collected from cameras and sensors. The data constitute the future period of the DT model and complement the full life-cycle of object development.

### 3.4. SoS Modelling and Application

Nostalgic life belongs to the SoS layer. However, SoS-layer DT modelling is not simply composed of system-layer DTs. The SoS layer is the integration of life cycles which are present in multiple scenes, and it is segmented based on lifestyles. SoS-layer modelling collects data from all aspects of the life cycle on the system layer and restores and processes data in a distributed manner (Qi et al., 2018). The life cycles in different scenes are connected by timeline correspondence.

In order to serve human emotions, a DT model of nostalgic life—integrating multiple nostalgic scenes—can be applied to situations such as museums and folklore tourism villages as a record of the regional history, which allows visitors to experience an inaccessible lifestyle that becomes the past and cannot be experienced anymore. In addition, if an individual has experienced many of the nostalgic scenes and restores the part of lost memories, an individual archive with exclusive memory can thus be formed. This paper focuses on the construction of DTs of nostalgic scenes and shelves the practice and verification of SoS.

## 4. Case Study: An Example of Rural Life in Shaanxi, China

Over the past 40 years of China’s development, approximately 500 million people have migrated from rural to urban areas, often facing psychological maladjustment due to lifestyle disparities. Young individuals relocating to cities for work have found emotional solace in memories of their hometowns, with nostalgia evolving into a ‘sense of belonging’. Two villages in Xi’an, Shaanxi province, which is part of China’s Guanzhong Plain, were selected as the target sites for the study. Geographically, the Guanzhong Plain, including parts of Shaanxi, Gansu, and Shanxi provinces, is characterised by its flat terrain and fertile soil, ideal for cultivating crops such as wheat and corn. Many residents have transitioned from rural to urban lifestyles due to either immigration or urban development. Rural life is marked by distinctive characteristics, such as agricultural cultural symbols, behaviours, and farmers’ attire. To verify the feasibility of the nostalgic scene design and the validity of the design principles, researchers selected “farm work” as the regional lifestyle of Xi’an, and identified the key FEATUREs (events and objects) that could evoke nostalgia as units. 

As shown in Table 1, the research’s outline comprises three steps: identifying FEATUREs, generating nostalgic scenes, and evaluating nostalgic scenes.

### 4.1. Identifying FEATUREs to Design Nostalgic Scenes

#### 4.1.1. Participants

Researchers conducted interviews with participants in Huangyan Village and Zhengjiazhuang Village, Shaanxi Province, China. Huangyan Village was an agricultural village 40 years ago, where some older residents previously worked as agricultural labourers, while the urbanisation of this city attracted a large working population from rural areas. Zhengjiazhuang Village, farther from the city, remains an agricultural village, with some elderly no longer able to work. 

The researchers randomly conducted initial interviews in both locations, using a combination of online and field-based methods. The first step involved screening the participants. If interviewees answered ‘yes’ to the first two questions, they were selected for the regional group; otherwise, they were assigned to the non-regional group. Interviewees were first asked if they had the same lifestyle (farm working in the field), and second, if they had been removed from that lifestyle. A total of 68 interviewees were screened as participants, with 34 in the regional group and 34 in the non-regional group. All participants lived in Xi’an and spoke Chinese as their first language. Participants in the regional group are now out of agricultural life, no longer engaged in productive labour or working in other industries outside the countryside. Their insights are used to screen for appropriate FEATUREs. In contrast, participants in the non-regional group come from areas outside the Guanzhong Plain. They may not have experienced farm work or have experienced it in their hometowns, and they all work and live in Xi’an city currently. The participants’ information is recorded in Table 2, including age, identity, and region.

#### 4.1.2. Obtaining FEATUREs and Recording Data

The researchers conducted semi-structured interviews lasting 10–30 min with each participant in the regional group, focusing on the topic of farm work. We asked the three questions listed in Table 1 to explore which FEATUREs, experiences, and changes could evoke nostalgia. Participants’ responses pertained to their decades-long life experiences or past customs, allowing them to convey their meanings on their own terms. This flexible interview style facilitated an open discussion, enabling participants to express their genuine thoughts.

Researchers counted the frequency of FEATUREs from the initial study, as shown in Table 2, indicating that participants share similar FEATUREs and that the same nostalgic scenes can emotionally resonate with them. The six most frequently occurring FEATUREs with more than 10 instances (wheat, harvesting wheat, livestock, carrying wheat, two-wheelers, and huts) were selected as key FEATUREs for reference. Participants’ general descriptions of FEATUREs were also encoded as data and logical rules. For example, some participants described the two-wheelers as “about two meters long, slightly wider than a human, parked at the edge of the field, with a donkey tethered to the side” and this language was used to extract data. As shown in Table 3, the model column represents the entity model of the objects and events, and the other data of key FEATUREs extracted from the interviews can be used to guide the combination and arrangement of FEATURE models. 

Following the preliminary interview, their contact information was collected, and a second interview was conducted at a later date.

### 4.2. Generating a Nostalgic Scene

#### 4.2.1. Co-Occurrence Analysis

**Figure 6:**
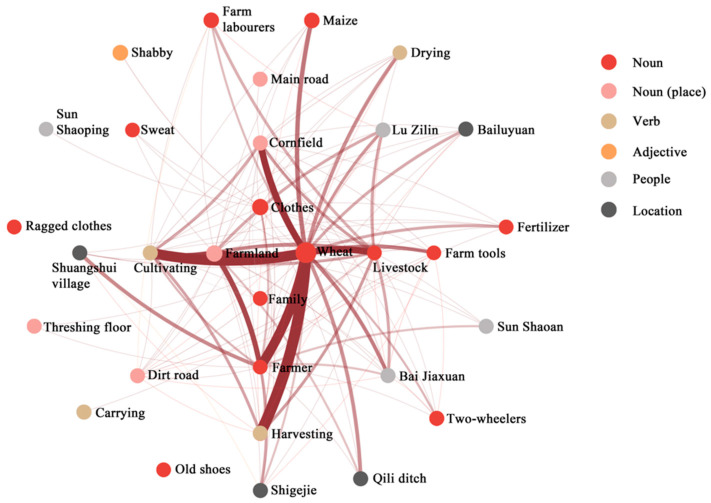
Co-occurrence analysis of the words in the three books.

**Figure 7:**
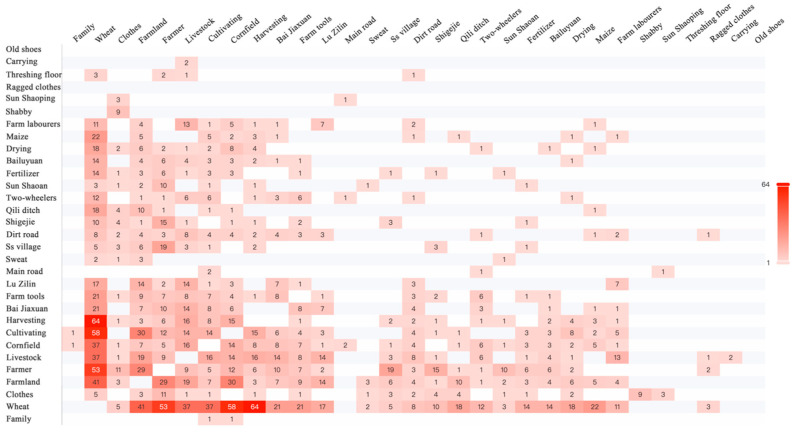
Co-occurrence matrix of words in the three books.

Co-occurrence analysis was employed to verify the correlation between key FEATUREs. On Douban, a popular Chinese book review website, we selected three highly praised realistic novels set in rural Shaanxi, *White Deer Plain*, *Ordinary World*, and *Shaanxi Opera*, as references for the lifestyle of farm work. Paragraphs depicting scenes and environments from these novels were extracted and consolidated into a single document. Word segmentation and co-occurrence analysis were conducted using Python 3.8, with synonym replacement initially performed to enhance the analysis. The results reveal the top 30 words with the highest frequency, excluding those with ambiguous meanings, such as “father” and “wife”. These words encompass nouns, verbs, adjectives, locations, and people. Figure 6 and Figure 7 illustrate the correlation strength and co-occurrence frequency, respectively. Each circle in Figure 6 represents a word, with a larger circle indicating a higher total co-occurrence value, signifying a more significant role for that word. Thicker lines between words denote a higher frequency of co-occurrence. The word “wheat” emerges as the most significant, co-occurring frequently with verbs like “drying”, “harvesting”, and “cultivating”, suggesting that activities like “harvesting wheat” hold meaning. The co-occurrence of “wheat” with nouns such as “farmer”, “livestock”, “farmland”, and “farm tools” is particularly high. All key FEATUREs, except for “hut”, exhibit substantial total co-occurrence values in Figure 6, indicating that these events and objects frequently co-occur, thereby validating their systematic co-existence in the same scene without discord. This confirms the reliability of the FEATUREs obtained through interviews.

#### 4.2.2. Unit-Layer Modelling

**Figure 8:**
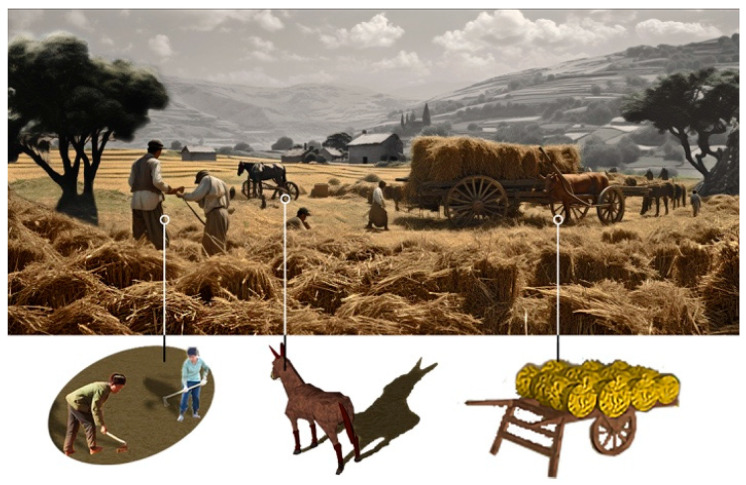
Nostalgic FEATUREs in Revit and Midjourney.

In this study, the nostalgic scene DT model was finally presented in the form of photos. Using Revit 2018 software, the unit layers were 3D modeled, arranged, and integrated into a DT model of the scene, following the rule model. Revit software was utilised for 3D modeling of the unit layers. The image generation tool Midjourney was employed for realistic rendering and generating photos from a particular perspective of the nostalgic scene DT model. Figure 8 illustrates a comparison of the FEATUREs’ 3D models in Revit and their visual representations in Midjourney.

Revit software was employed for the geometric modelling of five key FEATUREs (wheat, harvesting wheat, livestock, carrying wheat, and two-wheelers), with reference to data from Table 3. Tangible FEATUREs (two-wheelers and livestock) can be directly modelled by referencing dimensional data. Intangible FEATUREs (harvesting and carrying wheat) can be indirectly represented through the modelling of character actions and tools. In character modelling, we selected the 3D model of an elderly man in tattered clothes to better align with the memories described by the participants. To reduce file size, the 3D model of the background (wheat and farmland) can be replaced by 2D graphical models.

#### 4.2.3. System-Layer Modelling

**Figure 9:**
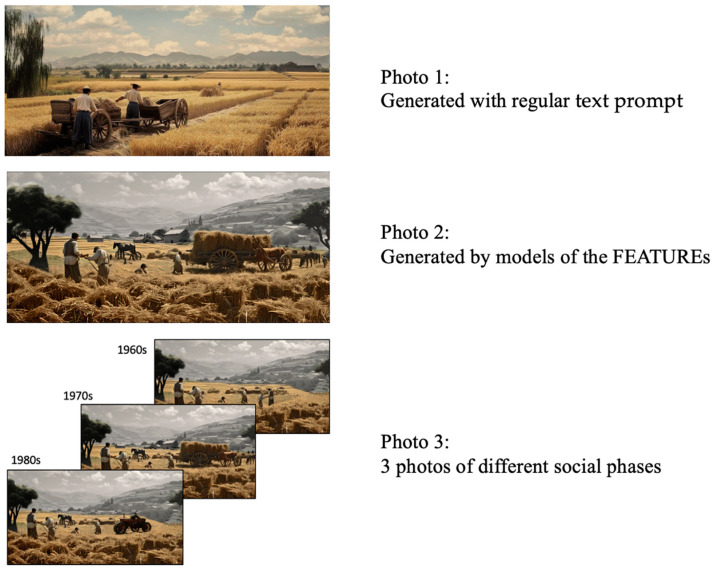
Three photos of the nostalgic scene by Midjourney.

As depicted in Figure 9, Midjourney was employed to generate three photos to investigate their impact on evoking nostalgia. To control for the visual features of each photo, all photos contained at least three of the following elements: field, people, and wheat, with the same size (2880 dpi × 1800 dpi).

Midjourney is an efficient Gen AI tool used as a demonstration. It was developed by an independent research laboratory, enabling users to generate realistic new images from text prompts. Midjourney has significant cultural and economic implications, particularly in creative design and education (Qi et al., 2018; Kastenholz et al., 2021). Its creation instructions specify the subject, setting, style, and scale of the desired image.

In contrast, a text prompt omitting FEATUREs was input, resulting in the image depicted in Photo 1 in Figure 9. The text prompt is “A nostalgic image of farmers toiling in the fields of Shaanxi province, China, in the 1970s, contains many elements of agriculture”. Photo 1 depicts a typical representation of a nostalgic scene.

Then, the geometric models of FEATUREs in Revit were exported as pictures, and input into Midjourney to generate photos combined with a text prompt. The text prompt is “A nostalgic image of farmers toiling in the fields of Shaanxi province, China, in the 1970s, front view, the farmers are using sickles to harvest wheat, Livestock and two-wheelers are at the edge of the field. There are stacks of wheat carried on the two-wheelers.” The geometric model of the FEATUREs in Revit can express size, material, and other details. Midjourney can preserve the original appearance of the input image (the FEATURE’s geometric model) and generate an image that is combined with the prompt. This means that the size contrast and physical relationships between FEATUREs remain consistent in the generated photos. The researchers also subjectively reviewed and verified the generated images to ensure that they accurately represent the physical model and do not overlap. One image with an appealing perspective was selected, as shown in Photo 2 in Figure 9. Photo 2 is a presentation form of the nostalgic scene DT model with a sense of systemicness.

Photo 2 in Figure 9 represents a nostalgic scene from a specific social phase. Similarly, a series of photos can be generated for different social periods, as illustrated by Photo 3 in Figure 9. Updating the FEATUREs allows for an updated scene, which in Midjourney can be easily generated by combining Photo 2 with text prompts. Taking the two FEATUREs (events) of “harvesting and carrying wheat” as an example, some participants recalled in the preliminary interview that, around the 1970s, farmers manually harvested wheat and used livestock to pull two-wheelers for transport, as depicted in Photo 2. Prior to that, around the 1960s, harvesting and carrying were performed manually; farmers did not breed livestock, and manufacturing was insufficient. By the 1980s, farmers rented mechanical equipment for harvesting and transport.

The text prompts are:

The 1960s: “A nostalgic image of farmers toiling in the fields of Shaanxi province, China, in the 1970s, front view, the farmers are using sickles to harvest wheat manually”, with Photo 2. The geometric models of two-wheelers and livestock were replaced by more labourer models.

The 1980s: “A nostalgic image of farmers toiling in the fields of Shaanxi province, China, in the 1970s, front view, the farmers are harvesting wheat with tractors, Livestock are at the edge of the field”, with Photo 2. The geometric models of two-wheelers were replaced by tractor models, and the number of livestock decreased. 

Photo 3 is composed of three generated images, representing a comprehensive presentation form of a nostalgic scene DT model across its full life-cycle.

### 4.3. Evaluating the Generated Nostalgic Scene

To validate the proposed method of nostalgic scene construction, a second interview was conducted among 34 participants in the regional group and 34 participants in the non-regional group. A perception experiment was set up with five different nostalgia perspectives (Kastenholz et al., 2021; White et al., 2020). The regional group was invited to observe Photos 1, 2, and 3, while the non-regional group was invited to observe Photos 2 and 3. After viewing each photo for five minutes, participants were invited to answer questions on a Likert scale from 1 (strongly disagree) to 5 (strongly agree). 

IBM SPSS 4.0 software is utilised to analyse the questionnaires for reliability. All 170 cases from 68 participants are valid, with a Cronbach’s alpha of 0.859 and a KMO value (Kaiser–Meyer–Olkin measure of sampling adequacy) of 0.799, indicating good reliability and validity. Factor analysis using principal component analysis reveals one component with a 64.834% variance percentage, confirming that the five questions reflect the same main factor: nostalgia.

Table 4 shows the descriptive statistics of the nostalgia condition. The regional group reported higher nostalgia towards Photo 2 than Photo 1, suggesting that systematic nostalgic scenes can make people perceive more authentic nostalgia. Some participants explained that in Photo 1 they only noticed nothing but people who carried wheat, and that the clothes of the characters, which are similar to those worn by workers rather than farmers, were strange to them, because the clothes were neat. The design of nostalgic scenes without guidance of systemicness may result in the inclusion of irrelevant objects in the scenes, thereby compromising the instant feeling of the scene and creating a sense of being out of place. Additionally, the regional group reports higher nostalgia towards Photo 3 than Photo 2, suggesting that nostalgic scenes with a full life-cycle can make people recall the historical evolution. Furthermore, 22 participants (65%) mentioned that multiple scenes of historical changes reinforced nostalgia more than a single static picture of a scene. The participants said, “Although these objects alone can evoke a bit of nostalgia for the past, when they are placed in the right context within the scene, it is not just the scene itself that becomes more real; it also makes me feel more nostalgic”. Some youngsters said, “When I was a child, I rarely participated in farm work, but it is very memorable because it was too hard.” Young people could not identify the three social phases in Photo 3 because different tools and equipment had co-occurred in the fields when they were children.

By comparing the evaluation of Photo 2 and Photo 3 by the regional and non-regional groups, it is proved that regional nostalgic scenes can only evoke nostalgic emotions of particular population groups. Especially in Q1, Q2, and Q4, there is a significant difference between the scores of the two groups. In light of the participants outside these regions, the presentation of scenes may not effectively evoke nostalgia due to different lifestyles. The non-regional group mentioned, “I have seen such a scene in the book, and it is not the same as growing rice in my countryside, but I think they are both drudgery. I think I would like to experience it if I had the chance.”

Both groups displayed different ratings for each photo in Q3. While some old and young people wanted to experience such a life of labour again, not all participants thought the former agrarian life was good. Some younger participants reflected that they did not like the agrarian lifestyle but would be willing to attempt it again without strain. Regarding the elderly who continue to reside in rural areas and no longer engage in labour due to poor health, they are deeply attached to and accustomed to agrarian life mentally, possessing a strong inner sense of belonging and a willingness to experience it again. Similarly, in Q5, the diverse evaluations of the regional group suggest that the participants are inevitably influenced by their familiarity with urban life. For the non-regional group, many young people are curious about farm work in Shaanxi, and they are willing to have short-term experiences. As for Q5, 79% of the participants in the non-regional group unanimously agreed that the scenes in the two photos did not evoke a sense of belonging.

People perceived stronger nostalgic feelings through systematic and full life-cycle nostalgic scenes than normally generated nostalgic scenes. The nostalgic feelings are stronger for locals than for outsiders, as the FEATUREs are regionally sampled. Hence, the DT design system of nostalgic scenes with three principles—regional, full life-cycle, and systematic—was verified/validated, which can be used to produce nostalgic scenes for integration into nostalgic life.

## 5. Discussion

Nostalgia design is abstract, complex, and personal. This study presents a DT design system of nostalgic scenes in a more systematic manner than existing vague and non-standardised approaches, enabling design for groups with similar lifestyles and emotions. DT-based nostalgic scene design exhibits strong regional, full life-cycle, and systematic features. 

The research focuses on DT modelling of nostalgic scenes. It validates the effectiveness of the SoS hierarchical DT modelling approach within nostalgic scenes through interviews with 68 participants across two groups. Modelling is performed hierarchically, from unit layer to system layer and then to SoS layer, in which data collections, model parameters, and logical relationships are established. By comparing the questionnaire results from three photos generated, which are created by Gen AI and provided to regional group participants, the effectiveness of systematicness and full life-cycle in the process of constructing digital twin models for nostalgic scenes can be proved. In the design of the nostalgic scenes, the details concerning the characters’ clothing slightly affect the participants’ perception of the instant feelings of the scene. By comparing the questionnaire results from the two groups of participants for the latter two photos, the effectiveness of regional characteristics in constructing digital twins for nostalgic scenes can be proved. Above all, however, in the nostalgia questionnaire, the answer to question c is less affected, and some non-regional participants who have not experienced a similar agricultural life expressed curiosity and anticipation for a given scene. All interviewees agree that the richer the relevant FEATUREs in the scene are, the faster and more obvious their nostalgia is triggered.

### 5.1. Limitations

The study’s limitations are as follows:

1. This article is not focused on the multi-sensory presentation form of nostalgic life, the establishment of associations in nostalgic scenes, and the monitoring of FEATUREs which will be complemented gradually afterward.
2. Due to the limitations of sites and devices, the research uses scene photos, which are generated by Gen AI, from different social phases to represent the nostalgic scene DT model. As a primary nostalgic trigger with regional, full-life-cycle, and systematic characters, this study does not realise the dynamic trend of the nostalgic scenes that change in real time.
3. In terms of DT modelling in the nostalgic scenes, some questions still need to be clarified. For example, nostalgic life involves social science and historical searches. It is hard for the programs in the devices to understand the logical rules that need to be considered during the modelling process. The rules even contain knowledge such as customs and taboos. Hence, it is inefficient to collect FEATURE data from designed interviews manually and then check whether the scenes logically conform. However, translating verbal elaborations into semantic expressions during modelling is also a key research priority. All of these aspects should be improved in the future.

### 5.2. Practical Implications

Different from DTs applied to industrial manufacturing and construction, the DT of a nostalgic scene primarily depicts an idealised scene within people’s minds. Although a community which is similar to this nostalgic scene may not exist, a harmonious construction of a nostalgic scene design can be realised through the combination of rich emotional experiences and associations. This logical and vast information shows a stronger effect on evoking people’s nostalgia. The DT system can be used in museums and other cultural venues and serves as a regional public memory for the masses. Such a system can serve migrant groups or the elderly, reduce their social pressure, and improve their emotional wellbeing. Managers use output devices to demonstrate and provide an immersive experience to visitors. Meanwhile, ordinary users can interact with the DT to delve into their inaccessible past experiences. The application of nostalgia design can be extended into the realms of venue design and personal digital archives.

(1) Venue design

Past experiences of life originally existed in physical space, but now, liberal economic forms have made nostalgia a consumable commodity, creating DT space to offer familiar experiences, consumption, and emotional space. Digital twins of nostalgic scenes are accessible to tourists in venues such as urban museums and folklore tourism villages, offering the chance to virtually experience destinations, objects, or phenomena that are otherwise inaccessible due to economic, geopolitical, or other constraints (Yao, 2020).

The systematic design of nostalgia is also evident in the rich sensory stimulation and authentic interactive experiences it provides. For instance, traditional arts and folklore performances highlight multisensory information through dialect, traditional motifs, and materials, which may qualify for cultural heritage status for protection and digitisation. Display technologies such as VR, AR, MR, WebGL, and holographic projection can replicate 3D models of physical FEATUREs and life videos. These technologies enable residents to experience the transformation from virtual to physical life (Sang, 2024). Digital human technology and generative AI enhance the authenticity of content and the engagement of interactive experiences. Future studies should investigate and quantify the effects of various sensory stimuli and levels of interaction on nostalgia.

(2) Personal Digital Archive

By recording and editing nostalgia scenes from a personal perspective, a low-cost personal digital archive of self-experience can be created. Although individuals cannot physically interact with virtual objects, their bodies remain the conduit for engagement. Their consciousness, emotions, and spirit are affected by the twin environment, allowing them to experience and revisit the past through personal digital archives, complementing the partial digital life archives of individuals with similar experiences (Tickner, 2016). The potential benefits of life archives include utilizing recorded events or social sharing scenes, supporting human memory, preserving the experiences of their families after their death, realizing a personal digital twin (PDT), forming a digital life file for individuals, and more. The realisation of a universal digital archive also hinges on the advancement of technologies like data storage, big data, and blockchain (Gonzaga, 2019).

## 6. Conclusions and Future Research

In this study, the focus lies on regional DT-based nostalgic scene design. After reviewing relevant literature and addressing the issue of nostalgia arising from pressure between rapid urban development and drastic lifestyle changes, the design principles of the nostalgic scenes are proposed: regional, full life-cycle, and systematic. Therefore, a DT system for regional nostalgic scenes is put forward, which is consistent with these principles. Based on SoS theory, a DT model of nostalgic scenes is constructed in this study. Nostalgic scenes are regarded as a model on the system layer, and related events and objects (regarded as unit layer models) are selected, collected, modelled, and combined into nostalgic scenes.

To verify the proposed system, a case study was implemented in rural Shaanxi Province and a classic nostalgic scene of farm work in the Guanzhong Plain was constructed. FEATUREs (objects and events) related to nostalgia and their development were collected and recorded manually from participants, so as to combine them into nostalgic scenes. The co-occurrence analysis of elements in local popular documentary literature verifies the correlation between events and objects in the scene. With FEATUREs and their evolution as input, Gen AI helps to generate photos from a particular perspective of a DT-based nostalgic scene in different generations. The results from the questionnaire of the three generated photos verify the design principles and demonstrate the effectiveness of the system of nostalgic scenes. Photos of nostalgic scenes can evoke memories of hard and poor labour among local participants, and they also repeatedly mention that the bittersweet past was mixed with drudgery. To some extent, it is also supported by Leunissen, who suggested that “nostalgia is ambivalent, and the positive tendency is stronger than the negative tendency” (Leunissen, 2023).

This study also proposes future work on the full life-cycle of nostalgic scenes. The current data of the rural village can be collected and processed on a sociological level through monitoring and forecasting. The DT system can also predict the population, vegetation cover, and desertification of the old community based on current static data. Multiple nostalgic scenes can be combined to restore the nostalgic life of residents, which helps people create individual digital life profiles. This study can reduce imbalance in regional development, improve emotional health, and expand understanding of DTs.

In the future, we will develop more interactive and multi-sensory presentation forms and more consecutive and dynamic nostalgic triggers, such as videos or 3D models of scenes. In addition, we will collect FEATURE data by taking existing villages as sample areas to more reliably support the dynamic changes of units within the nostalgic scenes. In the future questionnaire, we will choose more participants with different living backgrounds to conduct interviews and obtain a variety of nostalgic triggers.

## Figures and Tables

**Table 1:** Workflow.

Objectives	Interview/Work
Step 1: Preliminary interview—select regional participants to obtain FEATUREs	(1) Screening question: a. Have you experienced (or participated in) the specified life of farm work in Xi’an? b. Have you been removed from the specified life?
	(2) Information: a. Age (or age range) b. Identity information c. Region
	(3) Semi-structured interview: a. Which FEATUREs evoke nostalgia in you? b. What is your experience (or witness) with these key FEATUREs? c. How have they changed?
Step 2: Generate photos of the nostalgic scene with key FEATUREs	Statistics the frequency of FEATUREs, select key FEATUREs and verify their correlation to generate nostalgic scene photos in Revit and Midjourney.
Step 3: Second interview—evaluate the generated nostalgic scene	Likert scales:
	Q1. I can relate to the scene in the picture.
	Q2. I was impressed by the scene in the picture.
	Q3. I want to experience the scene in the picture again.
	Q4. The scenes in the picture evoke special memories for me.
	Q5. The scene in the picture makes me feel a sense of belonging in my heart.

**Table 2:** Profile of the 68 participants.

Participant Characteristics		N (%) Region	N (%) Non-Region
Sex	Male	17 (50)	17 (50)
	Female	17 (50)	17 (50)
Identity information	Elderly, still living in the countryside (≥60)	8 (24)	0 (0)
	Elderly, out of the countryside (≥60)	9 (26)	7 (21)
	Younger, used to work in agriculture (<60)	11 (32)	12 (34)
	Younger, used to experience or participate in agriculture (<60)	6 (18)	8 (24)
	Younger, has no contact with the countryside (<60)	0 (0)	7 (21)
Region	Shaanxi, China	28 (82)	0 (0)
	Henan/Gansu, China	6 (18)	0 (0)
	Other provinces (northern China)	0 (0)	8 (24)
	Other provinces (southern China)	0 (0)	26 (76)
Nostalgic FEATUREs (events or detailed objects)	Wheat (wheat crib)	26 (76)	/
	Harvesting wheat	21 (62)	/
	Livestock	17 (50)	/
	Carrying wheat	15 (44)	/
	Two-wheelers	14 (41)	/
	Hut	11 (32)	/
	Farm tools	8 (24)	/
	Ragged clothes	7 (21)	/
	Drying wheat	5 (15)	/
	Dirt road	3 (9)	/
	Trees between fields	2 (6)	/
	Old tractor	2 (6)	/
	Children playing under the tree	1 (3)	/

**Table 3:** The data of key FEATUREs by interview.

Key Features	Model	Size	Material	Rule/Knowledge
Wheat/wheat crib (object)	Wheat	Wheat: About 0.7 m high Wheat crib: About 0.5 m in diameter, 0.7 m high	Wheat	Wheat: on the ground Wheat crib: on the ground and two-wheelers
Harvesting wheat (event)	Farmers (bend over to reap), sickle	Sickle: About 0.3 m long and 3 cm in diameter	Wooden, metal	Near the wheat
Livestock (object)	Donkey (majority) cattle and horses (minority)	About 1.3 m in length, 1.3 m high	/	Near the two-wheelers (Two donkeys should not be tied too close together to avoid fighting)
Carrying wheat (event)	Farmers (holding), Wheat crib	Wheat crib: About 0.5 m in diameter, 0.7 m high	Wheat, wooden	/
Two-wheelers (object)	Two-wheelers	0.8 m wide, 2 m long and 0.6 m high	Wooden	At the edge of the field
Hut (object)	Hut	About 3 m in length and width, 2 m high	Thatch, mud	At the edge of the field

**Table 4:** Likert-scale statistical results.

	Regional Group						Non-Regional Group			
	Photo 1		Photo 2		Photo 3		Photo 2		Photo 3	
	M	SD	M	SD	M	SD	M	SD	M	SD
Q1.	3.41	0.925	3.97	0.758	4.24	0.741	2.38	1.074	2.59	0.988
Q2.	3.21	1.008	3.74	1.082	4.03	0.937	2.09	0.933	2.29	0.97
Q3.	3.26	1.082	3.32	1.036	3.56	1.05	2.82	1.359	2.68	1.224
Q4.	3.35	0.812	3.59	0.857	4.18	0.626	2.06	0.952	2.21	0.914
Q5.	3.12	1.2	3.26	1.136	3.79	1.008	1.41	0.609	1.35	0.544

Note: Q1–5 is shown in Table 1.

## Data Availability

Data are contained within the article.
